# Supplementary material for: The interactome of intact mitochondria by cross-linking mass spectrometry provides evidence for coexisting respiratory supercomplexes
Source: Mol Cell Proteomics. 2017 Dec 8;17(2):216–32. doi: 10.1074/mcp.RA117.000470 (PMC5795388; doi:10.1074/mcp.RA117.000470)
Supplement: Supplemental Data [file supp_RA117.000470_133922_0_supp_23978_fzffwf.zip › spectra_annotation/mito_spectra_annotation/233-1-12-1-3-1.pdf]

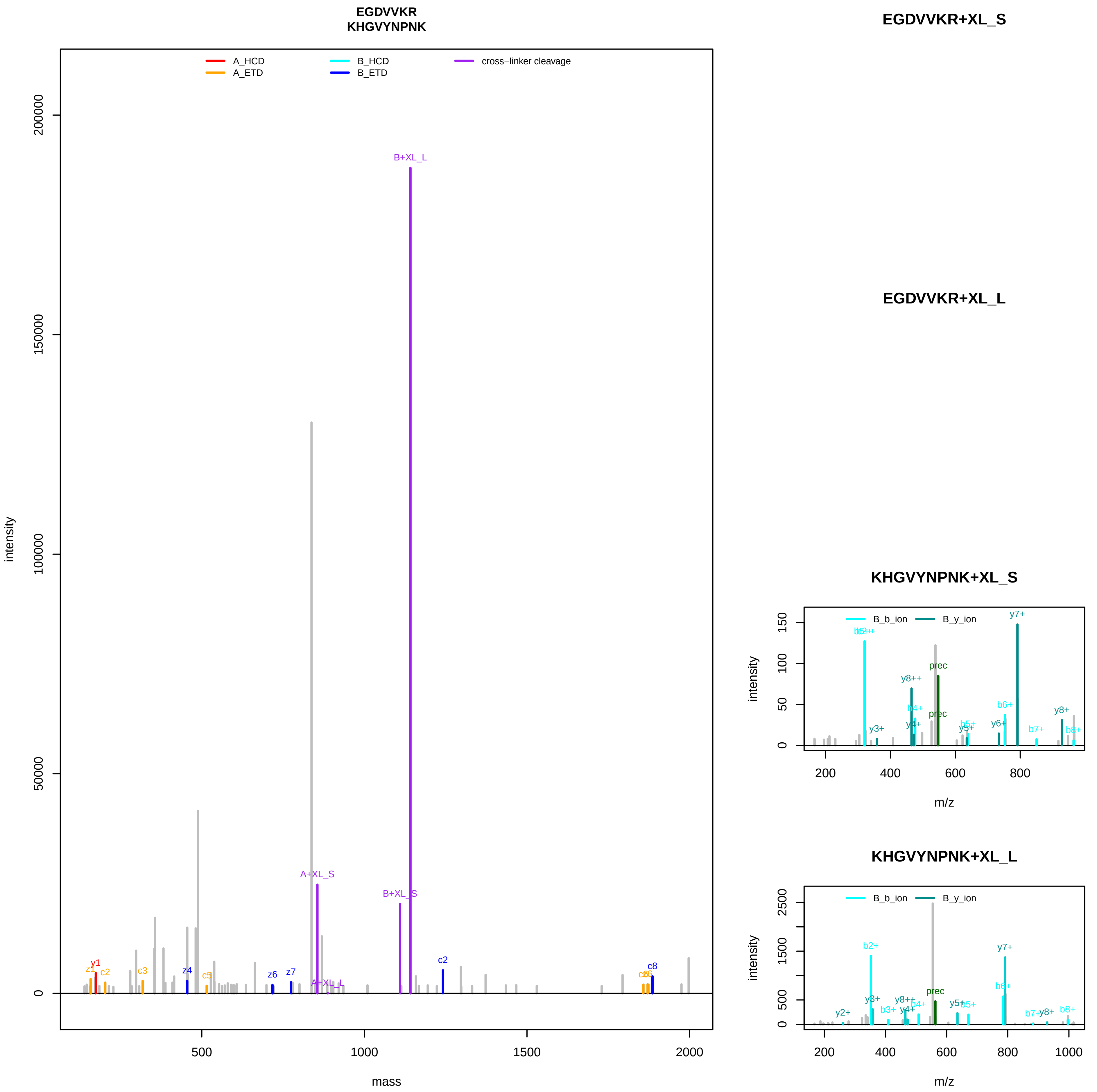
EGDVVKR
KHGVYNPNK
A_HCD
A_ETD
B_HCD
B_ETD
cross−linker cleavage
B+XL_L
A+XL_S
B+XL_S
A+XL_L
y1
z1
c2
c3
z4
c5
z6
z7
c2
c8
intensity
mass
0
50000
100000
150000
200000
500
1000
1500
2000
EGDVVKR+XL_S
EGDVVKR+XL_L
KHGVYNPNK+XL_S
B_b_ion
B_y_ion
y7+
prec
y8++
y3+
b4+
y4+
b5+
y5+
y6+
b6+
b7+
y8+
b8+
m/z
0
50
100
150
200
400
600
800
KHGVYNPNK+XL_L
B_b_ion
B_y_ion
b2+
y7+
b6+
y3+
y8++
prec
b4+
y5+
b5+
y2+
b3+
y4+
b7+
y8+
b8+
intensity
m/z
0
500
1500
2500
200
400
600
800
1000
